# IRF5 Is a Key Regulator of Macrophage Response to Lipopolysaccharide in Newborns

**DOI:** 10.3389/fimmu.2018.01597

**Published:** 2018-07-11

**Authors:** Anina Schneider, Manuela Weier, Jacobus Herderschee, Matthieu Perreau, Thierry Calandra, Thierry Roger, Eric Giannoni

**Affiliations:** ^1^Clinic of Neonatology, Department of Woman-Mother-Child, Lausanne University Hospital, Lausanne, Switzerland; ^2^Infectious Diseases Service, Department of Medicine, Lausanne University Hospital, Lausanne, Switzerland; ^3^Service of Immunology and Allergy, Department of Medicine, Lausanne University Hospital, Lausanne, Switzerland

**Keywords:** M1/M2 macrophages, newborns, innate immunity, interferon regulatory factor 5, monocytes, GM-CSF, LPS, tumor necrosis factor

## Abstract

Infections are a leading cause of mortality and morbidity in newborns. The high susceptibility of newborns to infection has been associated with a limited capacity to mount protective immune responses. Monocytes and macrophages are involved in the initiation, amplification, and termination of immune responses. Depending on cues received from their environment, monocytes differentiate into M1 or M2 macrophages with proinflammatory or anti-inflammatory and tissue repair properties, respectively. The purpose of this study was to characterize differences in monocyte to macrophage differentiation and polarization between newborns and adults. Monocytes from umbilical cord blood of healthy term newborns and from peripheral blood of adult healthy subjects were exposed to GM-CSF or M-CSF to induce M1 or M2 macrophages. Newborn monocytes differentiated into M1 and M2 macrophages with similar morphology and expression of differentiation/polarization markers as adult monocytes, with the exception of CD163 that was expressed at sevenfold higher levels in newborn compared to adult M1 macrophages. Upon TLR4 stimulation, newborn M1 macrophages produced threefold to sixfold lower levels of TNF than adult macrophages, while production of IL-1-β, IL-6, IL-8, IL-10, and IL-23 was at similar levels as in adults. Nuclear levels of IRF5, a transcription factor involved in M1 polarization, were markedly reduced in newborns, whereas the NF-κB and MAP kinase pathways were not altered. In line with a functional role for IRF5, adenoviral-mediated IRF5 overexpression in newborn M1 macrophages restored lipopolysaccharide-induced TNF production. Altogether, these data highlight a distinct immune response of newborn macrophages and identify IRF5 as a key regulator of macrophage TNF response in newborns.

## Introduction

Despite advances in perinatal care, neonatal infections remain a leading cause of mortality and morbidity worldwide (1–3). The high susceptibility to infection during the neonatal period has been linked to a developing immune system with a limited capacity to mount protective immune responses (4). Indeed, neonatal monocytes and dendritic cells (DCs) exposed to microbial products release reduced amounts of the proinflammatory and T_H_1-polarizing cytokines TNF, IFNγ, IL-1β, and IL-12p70 than adult cells, but similar or even higher levels of the T_H_17-polarizing and anti-inflammatory cytokines IL-6, IL-10, and IL-23 (5–7). Yet, uncontrolled inflammatory responses contribute to the pathogenesis of sepsis and septic shock and other conditions associated with adverse outcomes in newborns, such as necrotizing enterocolitis, bronchopulmonary dysplasia, and periventricular leucomalacia (8–11). Attempts at improving the outcome of neonatal sepsis through immune enhancing therapies including granulocyte colony-stimulating factor (G-CSF), granulocyte-macrophage colony-stimulating factor (GM-CSF), granulocyte transfusions, and intravenous immunoglobulins have only yielded a limited benefit (12–14). This underscores our incomplete understanding of how newborns respond to infections, and the need for new therapeutic approaches.

Tissue-resident macrophages are sentinel innate immune cells that display a spectrum of functions and produce a panel of cytokines that orchestrate innate and adaptive immune responses (15, 16). Macrophage activation and function are influenced by signals received from the local environment (17). The functional plasticity of macrophages has given rise to the notion of macrophage polarization, ranging from classically activated proinflammatory M1 macrophages to alternatively activated pro-resolving/anti-inflammatory M2 macrophages (18). The differentiation of monocytes into M1 macrophages is induced by GM-CSF, IFNγ, TNF, and bacterial lipopolysaccharide (LPS) (19–21). M1 macrophages are potent phagocytic cells that produce microbicidal molecules such as reactive oxygen and nitrogen species (ROS and NO) and TNF, IL-1β, IL-6, IL-12p70, and IL-23 (22, 23). In contrast, M-CSF, IL-4, IL-10, IL-13, adenosine, and steroid hormones induce the differentiation of monocytes into M2 macrophages (24). M2 macrophages are involved in resolving inflammation and promote tissue repair and homeostasis. M2 macrophages are characterized by the expression of scavenger receptors (CD36, CD163) and the production of high levels of IL-10 and low levels of TNF, IL-12p70, IL-23, ROS, and NO (25–27).

Monocyte to macrophage differentiation is controlled by the Janus-kinase/signal transducer and activator of transcription (JAK/STAT), MAP kinase (MAPK), and NF-κB pathways (28–31). These pathways activate suppressor of cytokine signaling (SOCS) and interferon regulatory factors (IRFs), leading to M1/M2 macrophage polarization (32, 33). In adults, IRF5, a downstream target of GM-CSF receptor (GM-CSFR), plays a critical role in driving macrophage polarization toward the M1 phenotype (23). However, the response of newborn macrophages to environmental signals driving M1 and M2 polarization and production of proinflammatory and anti-inflammatory cytokines is unknown.

Here, we report that in primary human monocytes exposed to GM-CSF, IRF5 was activated to a lower extent in newborns compared to adults during differentiation into M1 macrophages. Upon TLR4 stimulation, newborn M1 macrophages secreted lower levels of TNF compared to adult macrophages, while the production of other cytokines was not affected. Overexpression of IRF5 in newborn macrophages restored TNF production, suggesting a key role of IRF5 in shaping the distinct immune response of newborn macrophages.

## Materials and Methods

### Subjects and Source of Blood Samples

**Figure 1:**
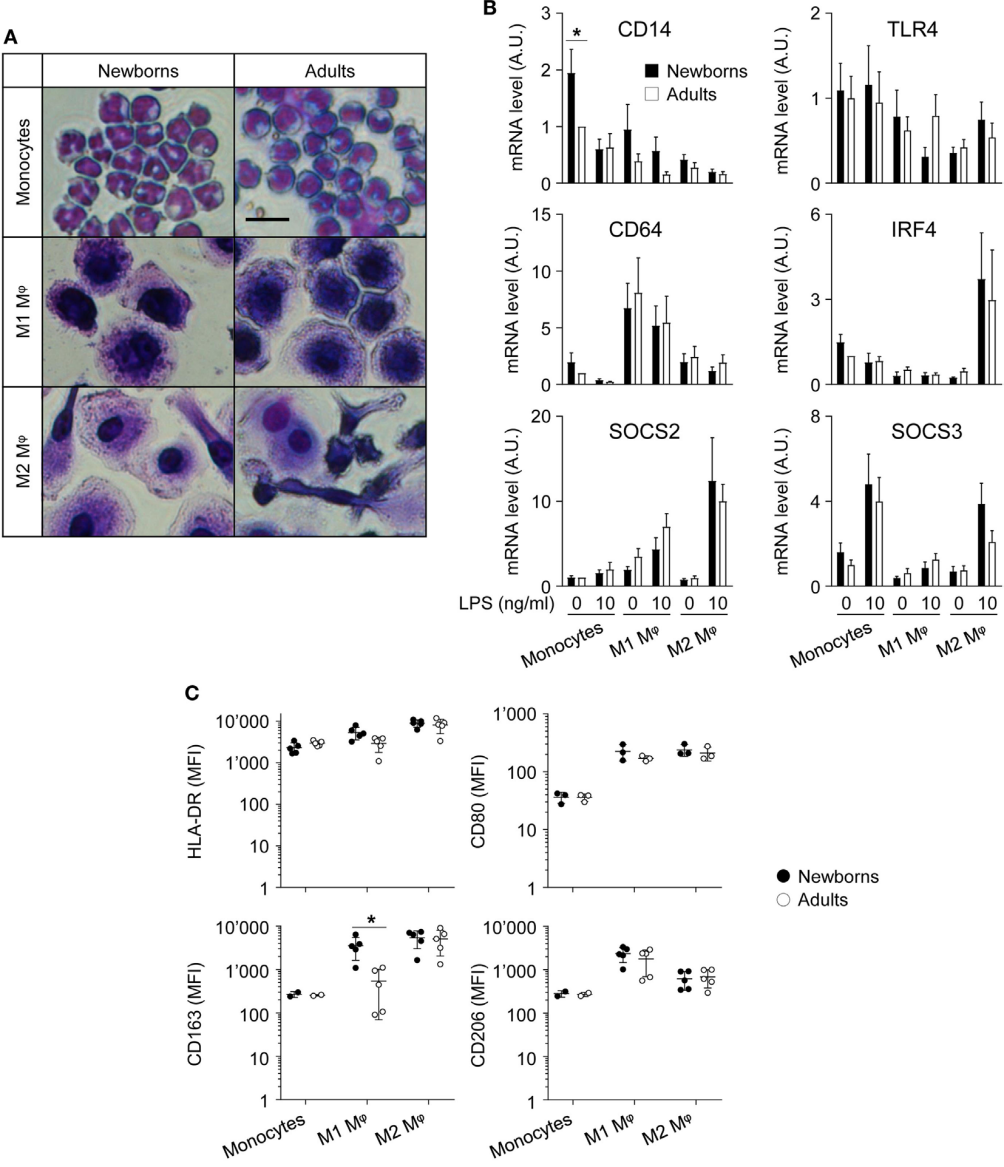
Adult and newborn monocytes differentiate into M1 or M2 macrophages following exposure to GM-CSF or M-CSF. Freshly isolated monocytes were cultured for 7 days with GM-CSF and M-CSF (50 ng/ml) to induce M1 and M2 macrophages. **(A)** Hematoxylin and eosin staining of monocytes and M1 and M2 macrophages. Scale bar = 30 µm. Data are representative of results obtained from five newborns and five adults. **(B)** CD14, TLR4, CD64, SOCS2, and IRF4 mRNA expression levels in newborn (black bars) and adult (white bars) monocytes and M1 and M2 macrophages were measured by RT-PCR. Data are means ± SEM from eight newborns and eight adults. **(C)** HLA-DR, CD80, CD163, and CD206 mean fluorescence intensity in newborn (black circles) and adult (white circles) monocytes and M1 and M2 macrophages was analyzed by flow cytometry in three to five healthy newborns and adults. Each dot represents one subject. CD163 and CD206 were not detected in monocytes from 2/5 newborns and 2/5 adults. Means ± SEM are presented. *P < 0.05.

**Figure 2:**
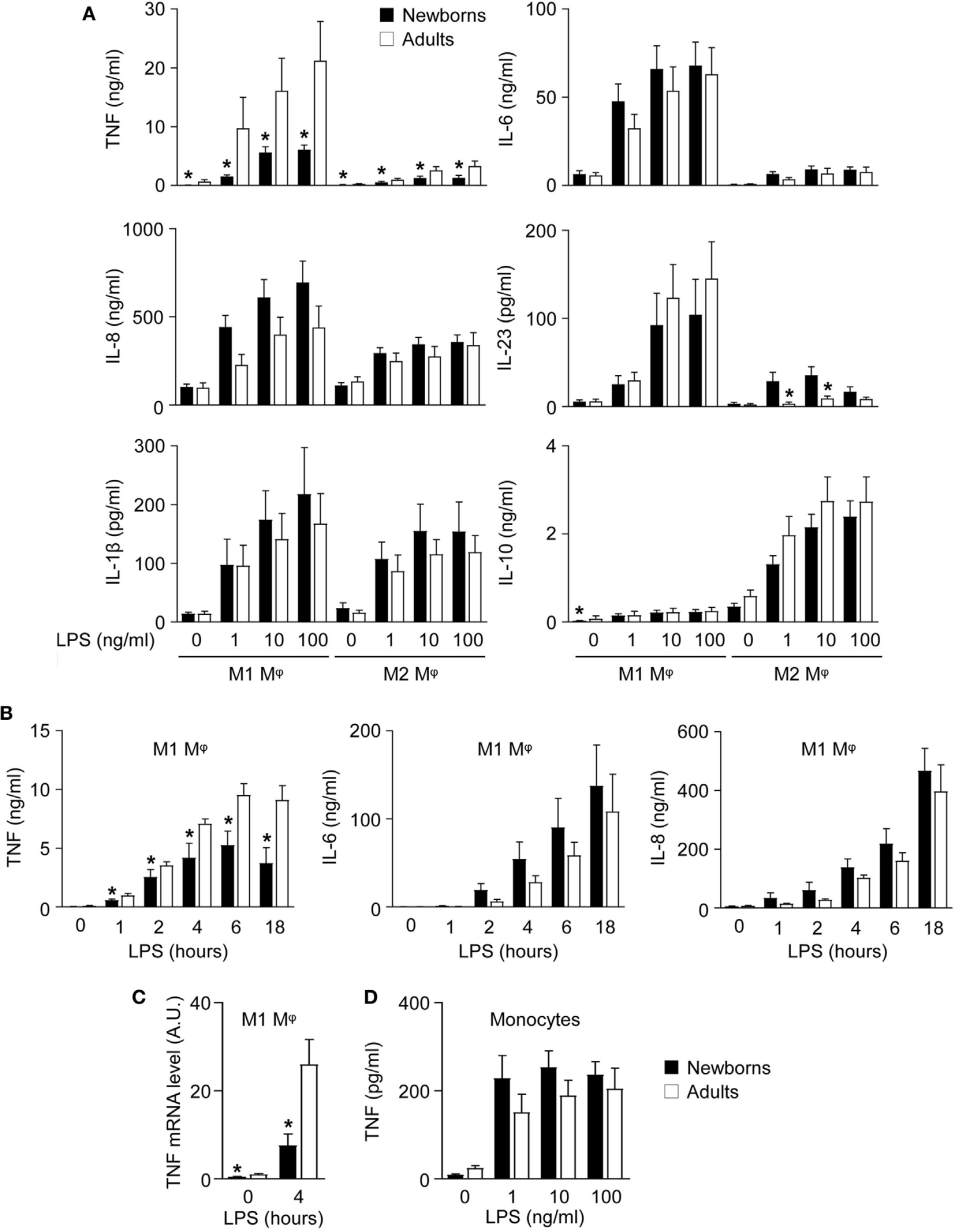
Newborn M1 macrophages secrete reduced amounts of TNF after lipopolysaccharide (LPS) stimulation. Newborn (black bars) and adult (white bars) monocytes were cultured for 7 days with GM-CSF (50 ng/ml) and with M-CSF (50 ng/ml) to induce M1 **(A–C)** and M2 macrophages **(A)**. Macrophages **(A–C)** and monocytes **(D)** were stimulated with 0–100 ng/ml LPS. **(A)** Cytokine levels were measured in cell culture supernatants collected after 20 h. Data are means ± SEM from 20 (TNF, IL-6, IL-8, IL-1β, and IL-10) or 10 (IL-23) newborn and adult subjects. **(B)** TNF, IL-6, and IL-8 levels were measured in cell culture supernatants collected after 0–18 h. Data are means ± SEM from eight newborns and six adults. **(C)** TNF mRNA expression levels in M1 macrophages exposed for 0 and 4 h to 100 ng/ml LPS were measured by RT-PCR. Data are means ± SEM from 10 newborns and 9 adults. **(D)** TNF concentrations in cell culture supernatants of monocytes exposed for 20 h to 0–100 ng/ml LPS. Data are means ± SEM from 20 newborns and 10 adults. *P < 0.05.

**Figure 3:**
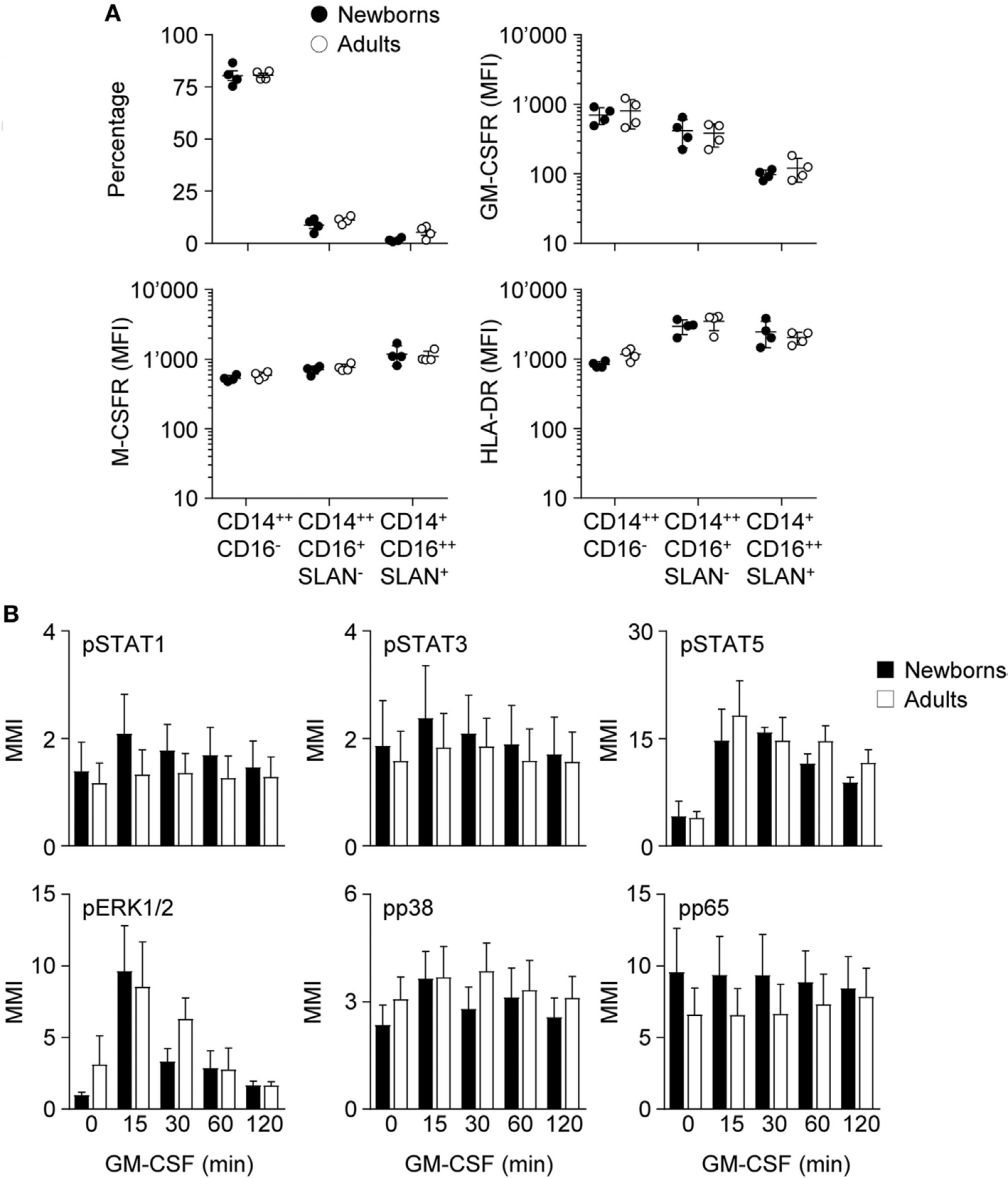
Proportions of monocyte subpopulations, expression of GM-CSF receptor (GM-CSFR), M-CSFR and HLA-DR and activation of intracellular signaling pathways by GM-CSF in newborn and adult monocytes. **(A)** Percentages of classical CD14^++^CD16^−^, intermediate CD14^++^CD16^+^SLAN^−^, and non-classical CD14^+^CD16^++^SLAN^+^ subpopulations and mean fluorescence intensity of GM-CSFR, M-CSFR, and HLA-DR in newborn (black circles) and adult (white circles) monocytes were determined by flow cytometry. Each dot represents one healthy subject. Means ± SEM are depicted. **(B)** Nuclear levels pSTAT1, pSTAT3, pSTAT5, pp38, pERK, and pNF-κBp65 in newborn (black bars) and adult (white bars) monocytes exposed for 15–120 min to 50 ng/ml GM-CSF were analyzed by CyTOF. Mean magnetic intensities were determined. Data are means ± SEM from six newborns and six adults.

**Figure 4:**
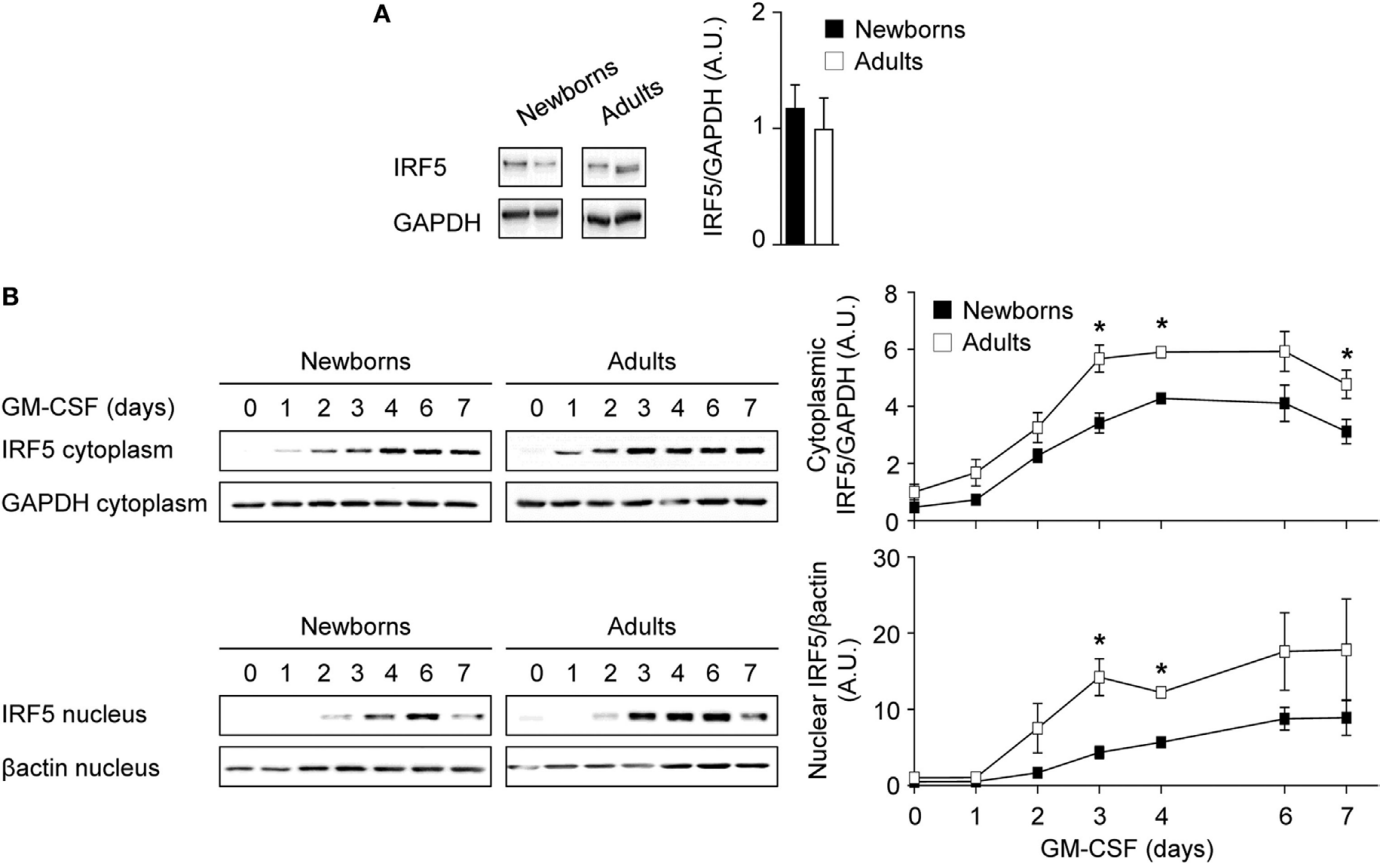
Reduced cytoplasmic and nuclear IRF5 levels during GM-CSF-induced monocyte to macrophage differentiation in newborns. **(A)** Intracellular IRF5 levels in freshly isolated newborn (black bars) and adult (white bars) monocytes. Data are means ± SEM from 10 newborns and 10 adults. **(B)** Cytoplasmic and nuclear IRF5 levels in newborn (black) and adults (white) monocytes cultured for 0–7 days with 50 ng/ml GM-CSF. IRF5 levels were analyzed by Western blotting (left panels) and quantified by imaging (right panels). Data are means ± SEM from nine newborns and nine adults. *P < 0.05.

**Figure 5:**
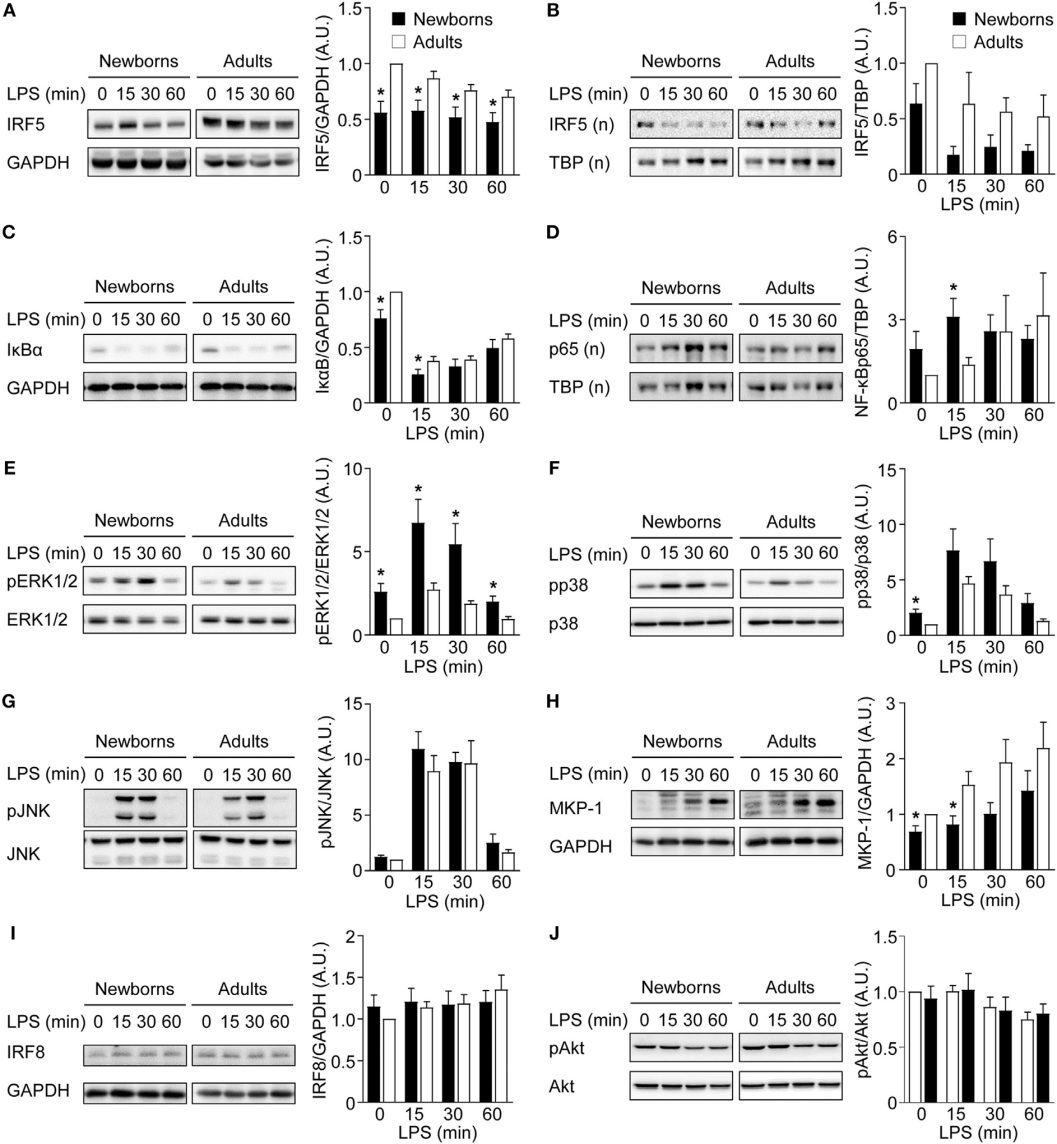
Reduced IRF5 expression levels in newborn M1 macrophages. Newborn (black bars) and adult (white bars) M1 macrophages were stimulated with 100 ng/ml lipopolysaccharide for 0–60 min. IRF5 **(A,B)**, IκBα **(C)**, NF-κBp65 **(D)**, phosphorylated and total ERK1/2 **(E)**, p38 **(F)** and JNK **(G)**, MAP kinase phosphatase-1 **(H)**, IRF8 **(I)**, and phosphorylated and total Akt **(J)** were analyzed by Western blotting using total **(A,C,E–J)** or nuclear **(B,D)** cellular extracts and quantified by imaging. Values were normalized to those obtained from resting adult M1 macrophages set at 1. Data are means ± SEM from 8 to 10 newborns and 8 to 10 adults. *P < 0.05.

**Figure 6:**
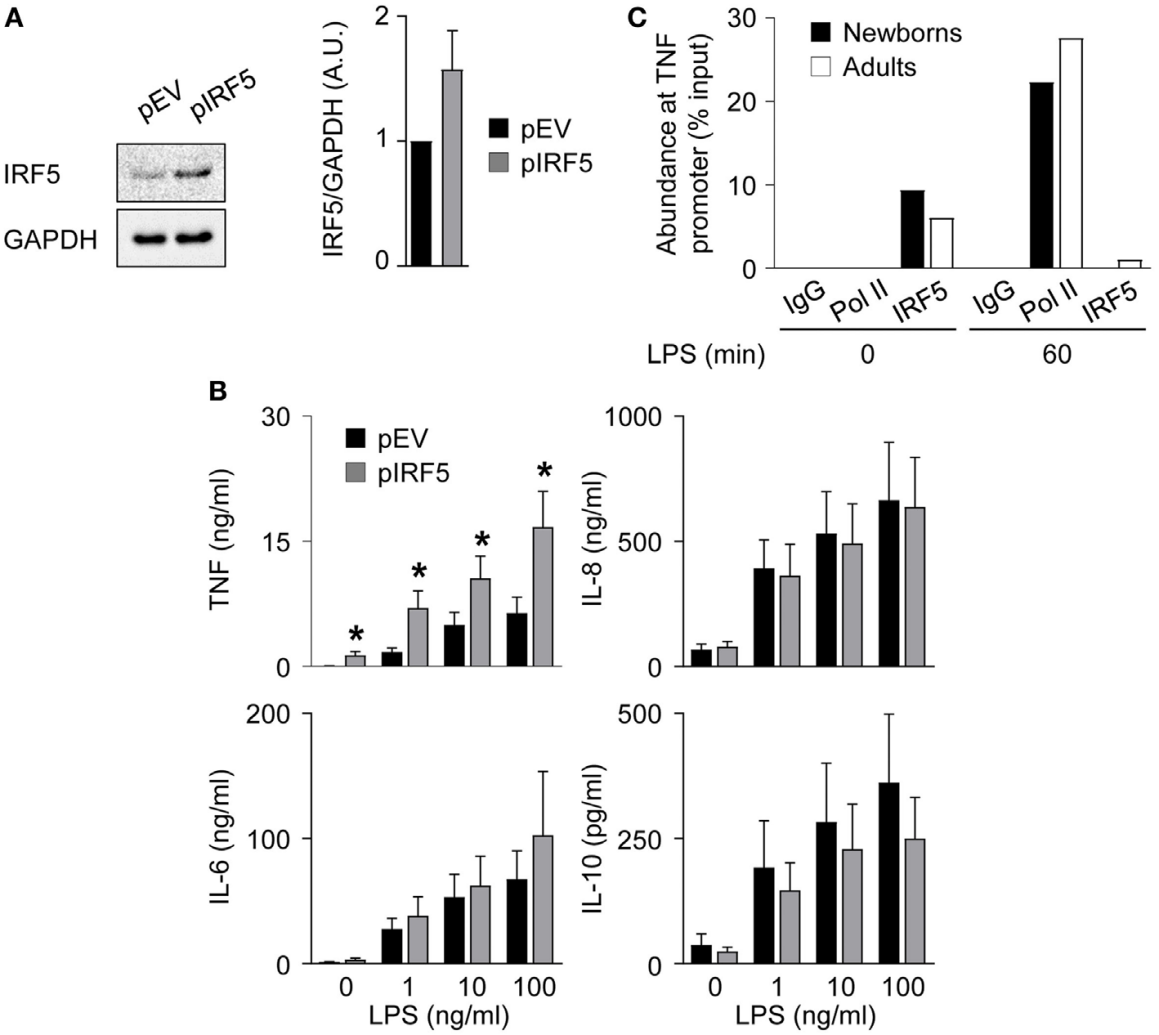
IRF5 overexpression restores TNF secretion by newborn M1 macrophages. Newborn M1 macrophages were transduced with empty control (pEV) (black bars) and IRF5-expressing (gray bars) adenoviral vectors **(A,B)**. **(A)** IRF5 levels were analyzed by Western blotting (left panel) and quantified by imaging (right panel). **(B)** Newborn M1 macrophages were stimulated with 0–100 ng/ml lipopolysaccharide (LPS). TNF, IL-6, IL-8, and IL-10 concentrations were measured in cell culture supernatants collected after 20 h. Data are means ± SEM from 20 (TNF) or 10 (IL-6, IL-10, and IL-8) newborns. *P < 0.05. **(C)** The recruitment of RNA polymerase II (Pol II) and IRF5 to the TNF promoter in M1 macrophages before and 1 h after stimulation with 100 ng/ml LPS was assessed by chromatin immunoprecipitation. Data from one experiment representative of two experiments are presented as the percentage input relative to genomic DNA set at 100%.

Umbilical cord blood was collected after delivery of the placenta of 91 healthy term neonates. Peripheral blood was obtained from 71 healthy adult volunteers (age 18–65 years). Monocytes and macrophages from the same subjects (20 newborns and 20 adults) were used for the experiments reported in Figures 1B and 2A,C,D. Macrophages from the same subjects (10 newborns and 10 adults) were used for the experiments reported in Figures 2B and 5A–J. Different sets of newborn and adult donors were used for every other Figures 1A,C, 3A,B, 4A,B, 5A–J, and 6A–C. Blood was collected in heparinized tubes (10 U/ml). Our study was approved by the Cantonal Human Research Ethics Committee of Vaud (CER-VD, Lausanne, Switzerland).

### Cells and Reagents

Mononuclear cells were isolated by Ficoll Hypaque (GE Healthcare) gradient density centrifugation. Monocytes were extracted from blood mononuclear cells by positive selection using magnetic microbeads coupled to anti-CD14 antibodies (Miltenyi Biotec) (34–36). Purity assessed by flow cytometry was >95%. Viability determined by trypan blue exclusion was >95%. Monocytes were cultured in RPMI medium 1640 supplemented with 10% (vol/vol) FCS (GE Healthcare) and GM-CSF (50 ng/ml) (Peprotech) or M-CSF (50 ng/ml) (Peprotech) for 1 week to induce M1 or M2 macrophages, respectively. Ultrapure *E. coli* O111:B4 LPS was purchased from List Biological Laboratories. Polyclonal and monoclonal antibodies (pAbs and mAbs) used for flow cytometry, Western blotting and cytometry by time of flight (CyTOF) are described in Table S1 in Supplementary Material. Unless specified otherwise, all other reagents were obtained from Sigma-Aldrich.

### RNA Analyses

RNA was extracted, reverse transcribed, and used in real-time PCR as described (37). The primers (5′–3′ sequences, sense and antisense) used for amplification were: HPRT, GAACGTCTTGCTCGAGATGTG and CCAGCAGGTCAGCAAAGAATT; CD14, CGCCCTGAACTCCCTCAAT and CTTGGCTGGCAGTCCTTTAGG; TLR4, AGTTTCCTGCAATGGATCAAGG and CTGCTTATCTGAAGGTGTTGCAC; CD64, TGCCACAGAGGATGGAAATG and CTGGAGGCCAAGCACTTGA; IRF4, AATCCTCGTGAAGGAGCTGA and GTAGATCGTGCTCTGGCACA; SOCS2, GGATGGTACTGGGGAAGTATGACTG and AGTCGATCAGATGAACCACACTGTC; SOCS3, GCTCCAAGAGCGAGTACCAG and CTGTCGCGGATCAGAAAGGT; TNF, CAGAGGGCCTGTACCTCATC and GGAAGACCCCTCCCAGATAG. Gene-specific expression was normalized to the expression of HPRT and was expressed in arbitrary units (A.U.).

### Flow Cytometry Analyses

Mononuclear cells and macrophages were stained using mAbs (Table S1 in Supplementary Material) as described (38). Thirty thousand events were acquired with a LSR-II flow cytometer (BD Biosciences). Data were analyzed using the BD FACSDiva™ software (BD Biosciences).

### Cytokine Measurements

Cytokine concentrations in cell-culture supernatants were measured by ELISA (BD Biosciences, for TNF, IFNγ, IL-1β, IL-6, IL-8, and IL-10) or by the Luminex technology (Affymetrix eBioscience, for IL-12p70, IL-20, IL-23, and IL-27).

### CyTOF Analyses

Monocytes were exposed for 0, 15, 30, 60, or 120 min to GM-CSF and fixed with formaldehyde at a final concentration of 1.5%. Cells were stained using an anti-CD14 mAb conjugated with the Fluidigm MaxPar conjugation kit (Fludigm). Cells were washed with Cell Staining Media and PBS, fixed with 2% formaldehyde, and bar-coded using Scn-Bn-EDTA-palladium barcode reagents (39). After barcoding, cells were pooled, permeabilized for 30 min at −20°C using 100% methanol, washed twice with 6 ml Cell Staining Media containing 0.3% saponin, and incubated for 30 min with mAbs directed against intracellular targets. Finally, cells were incubated overnight at 4°C in intercalation solution (PBS, 0.3% saponin, 1% formaldehyde, 125 nM Cell-ID Intercalator-Ir, Fluidigm) before acquisition on a CyTOF 1 upgraded to a CyTOF 2. Individual data files were concatenated, normalized, and deconvoluted as described (40) and were analyzed using Cytobank (Cytobank Inc.).

### Western Blot Analyses

Whole cellular extracts and cytoplasmic and nuclear extracts were prepared as described previously (34). Equal amounts of protein extracts were electrophoresed through SDS/PAGE. Proteins were transferred onto nitrocellulose membranes (Schleicher and Schuell). Membranes were incubated with Abs (listed in Table S1 in Supplementary Material) directed against NF-κBp65, IκBα, total and phosphorylated p38, ERK1/2, and JNK MAPKs, MAP kinase phosphatase-1 (MKP-1), IRF5, IRF8, total and phosphorylated Akt, GAPDH, β-actin, and TATA-binding protein. After washing, membranes were incubated with horseradish peroxidase-conjugated secondary Abs (Pierce Biotechnology Inc.). Signals were revealed using enhanced chemiluminescence detection (GE Healthcare). Images were recorded using a Fusion Fx system (Viber Lourmat).

### Adenovirus Transduction

IRF5-encoding and control empty adenoviral vectors (Applied Biochemical Materials Inc.) were amplified in HEK-293 cells (ATCC CRL-1573) and stored at −80°C in 10% glycerol. Macrophages were transduced with the adenoviral preparations (50 µl for 10^5^ cells, 1 ml for 2.5 × 10^6^), and used 24 h later for functional studies.

### Chromatin Immunoprecipitation (ChIP) Assay

Chromatin immunoprecipitation analyses were performed according to the manufacturer’s recommendations (MAGnifiy Chromatin Immunoprecipitation System, Thermo Fisher). Briefly, 1 × 10^6^ M1 macrophages were fixed with 1% formaldehyde. Chromatin was sheared by 16 cycles of 30-s pulse/30-s rest with an amplitude of 14% using an Ultrasonic Liquid Processor (Branson). Chromatin was incubated overnight at 4°C with 5 µg of antibodies directed against IRF5 (Cell Signaling Technology), or RNA polymerase II (Pol II, Table S1 in Supplementary Material), or with control IgGs (provided in the kit). Real-time PCR was performed with a 7500 Fast Real-Time PCR System using the SYBR Kapa Fast Mix (Sigma-Aldrich). The following sense and antisense primers (5′–3′ sequences) were used for amplification: TNF, TGCTTGTTCCTCAGCCTCTT, and TCACCCATCCCATCTCTCTC.

### Statistical Analyses

Statistical analyses were performed using PRISM (Graphpad Software Inc.). Data are expressed as means ± SEMs. Comparisons between the different groups were performed by two-two-tailed *t* tests. Findings were considered statistically significant when *P* < 0.05.

## Results

### Newborn and Adult Monocytes Differentiate into M1 and M2 Macrophages in Response to GM-CSF and M-CSF

The differentiation and polarization of freshly isolated newborn and adult monocytes into M1 and M2 macrophages following 7 days of culture with recombinant GM-CSF and M-CSF were analyzed by hematoxylin and eosin staining (Figure 1A) and by measuring the expression of maturation/differentiation markers by RT-PCR (CD14, CD64, SOCS2, SOCS3, and IRF4; Figure 1B) and flow cytometry (HLA-DR, CD80, CD163, CD206; Figure 1C). Monocytes and M1 macrophages from healthy term newborns and adult volunteers showed a round shape, while M2 macrophages displayed a more elongated shape, consistent with the expected phenotype (41, 42). No difference in morphology and viability (91 ± 1 versus 95 ± 1% and 91 ± 1 versus 94 ± 1% for newborn and adult M1 and M2 macrophages, respectively) was noticed between newborn and adult cells.

CD14 mRNA levels were higher (2.1- to 4.7-fold) in monocytes than in macrophages (Figure 1B), as anticipated (43). Unexpectedly, CD14 was more expressed (1.9-fold) in newborn than in adult monocytes. When compared to monocytes, TLR4 was expressed at lower levels in M2 macrophages (2.4- to 3.2-fold), while the M1 marker CD64 was enriched (5.2- to 8.1-fold) in M1 macrophages, and the M2 markers SOCS2 and IRF4 were enriched (5.1- to 8.1 and 3.6- to 4.8-fold) in LPS-stimulated M2 macrophages (44–47). SOCS3, a gene implicated in the repression of the M1 phenotype (48), was expressed at lower levels in M1 macrophages than in monocytes. Newborns and adult cells expressed similar levels of TLR4, CD64, SOCS2, SOCS3, and IRF4.

HLA-DR, CD80, CD163, and CD206 were expressed at similar levels by newborn and adult monocytes (Figure 1C). The mean fluorescence intensity (MFI) of each of the molecules increased, albeit to different extents, in M1 and M2 macrophages (MFI fold increase versus monocytes: HLA-DR: 2.3–3.9; CD80: 6.2–6.6; CD163: 33–50; CD206: 5.5–16.5). HLA-DR and CD80 MFI were similar in newborn and adult M1 and M2 macrophages. CD163 was previously reported as an M2 marker in adults (41, 49, 50). However, in newborns, CD163 MFI strongly increased in both M1 and M2 macrophages compared to monocytes (33- and 50-fold). CD206, an M2 polarization marker at the transcript level (27), was more expressed in GM-CSF than M-CSF-derived macrophages by flow cytometry (41, 49). Accordingly, CD206 MFI was higher in newborn and adult M1 macrophages than in M2 macrophages, without noticeable difference of expression between newborns and adults. Overall, following exposure to GM-CSF and M-CSF, newborn monocytes differentiated into cells adopting morphological features and expressing markers of M1 and M2 macrophages similar to adult cells, with the exception of CD163 that was expressed at higher levels in newborn than adult M1 macrophages.

### TLR4-Mediated TNF Secretion Is Selectively Reduced in Newborn M1 Macrophages

Functional studies were performed to compare the capacity of newborn and adult M1 and M2 macrophages to secrete proinflammatory and anti-inflammatory cytokines in response to TLR4 stimulation. In response to LPS, newborn and adult M1 macrophages secreted higher levels of TNF, IL-6, IL-8, and IL-23 and lower levels of IL-10 than M2 macrophages (Figure 2A). IL-1β secretion was similar between M1 and M2 macrophages. IFNγ, IL-12p70, IL-20, and IL-27 were undetectable.

Interestingly, newborn M1 macrophages secreted 3- to 6-fold less TNF (6.1 versus 21.2 ng/ml using 100 ng/ml LPS), while they produced IL-1β, IL-6, IL-8, IL-10, and IL-23 in the same range as adult M1 macrophages. Reduced TNF secretion was detected as early as 1 h following LPS stimulation (Figure 2B) and was associated with lower TNF mRNA expression in newborn M1 macrophages (Figure 2C).

We then evaluated whether the diminished TNF secretion by newborn M1 macrophages was also present in monocytes (Figure 2D). LPS-induced TNF secretion was much lower in monocytes than in macrophages and was similar in newborn and adult monocytes. These findings suggested that GM-CSF triggered a different response in newborn and adult cells resulting in a specific reduction of TNF production by newborn M1 macrophages. This was unlikely to be due to gender differences, as macrophages from males released similar amounts of TNF compared to macrophages from females, both in newborns and adults.

### Reduced IRF5 Activation During Monocyte to Macrophage Differentiation in Newborns

As a first approach to decipher the impact of GM-CSF on macrophage differentiation, we analyzed the expression CSF receptors and the activation of downstream signaling pathways in monocytes. Classical (CD14^++^CD16^−^), intermediate (CD14^++^CD16^+^SLAN^−^), and non-classical (CD14^+^CD16^++^SLAN^+^) monocyte subsets (51, 52) were equally distributed in newborns and adults and expressed similar levels of GM-CSFR, M-CSFR, and HLA-DR (Figure 3A; Figure S1 in Supplementary Material).

Binding of GM-CSF to the GM-CSFR initiates the JAK/STAT, MAPK, and NF-ĸB intracellular signaling pathways and activates IRF5 in DCs (53). The pathways activated by GM-CSF in newborn and adult monocytes were investigated by mass cytometry. Exposure of newborn and adult monocytes to GM-CSF increased the phosphorylation of STAT5 (3.5- to 4.6-fold) and ERK1/2 (2.8- to 9.9-fold), but not that of STAT1, STAT3, p38, and NFĸBp65 (Figure 3B). No difference was detected between newborns and adults.

IRF5 is a downstream target of GM-CSFR signaling and plays a key role in M1 polarization (23). IRF5 is activated by phosphorylation, leading to its dimerization and nuclear translocation to promote the expression of immune response genes (54). Intracellular levels of IRF5 were similar in newborn and adult monocytes (Figure 4A). We then quantified IRF5 in cytoplasmic and nuclear fractions obtained from monocytes exposed for 0–7 days to GM-CSF (Figure 4B). Cytoplasmic levels of IRF5 started to rise at day 1, peaked at day 3–6, and declined at day 7, while nuclear levels of IRF5 increased from day 2 to day 7. Cytoplasmic levels of IRF5 were 1.4- to 2.3-fold higher in adults than in newborns at days 1–7, while nuclear levels were 2.0- to 4.5-fold higher in adults from day 0 to day 7. Of note, IRF5 was detected at 15–20 lower levels in the nucleus than in the cytoplasm at day 7. Thus, during GM-CSF-induced monocyte to M1 macrophage differentiation, IRF5 was expressed at lower levels and translocated to the nucleus to a lower extent in newborn than in adult cells, a difference that might well explain the reduced expression of TNF in newborn M1 macrophages.

### Reduced IRF5 Expression in Newborn M1 Macrophages

To further characterize the mechanisms underlying M1 macrophage polarization, the expression of IRF5 and IRF8, another transcription factor implicated in M1 polarization (55), and the activation of NF-κB, MAPK, and Akt signaling pathways were analyzed in GM-CSF-induced M1 macrophages exposed to LPS for 0, 15, 30, and 60 min. Cytosolic IRF5 levels were lower in newborn than in adult M1 macrophages before and following LPS exposure (Figure 5A). Nuclear IRF5 levels decreased following LPS stimulation and were lower in newborns than in adults at all time points, although differences were not statistically significant (Figure 5B). Newborn M1 macrophages expressed lower levels of cytosolic IκBα and higher levels of nuclear NF-ĸBp65 before and 15 min after LPS stimulation (Figures 5C,D). Phosphorylation of ERK1/2 and p38, but not of JNK, was higher in newborn M1 macrophages at baseline and upon LPS stimulation (Figures 5E–G). In line with these findings, expression of MKP-1/dual specificity phosphatase (DUSP1), a DUSP that inactivates ERK1/2 and p38, was reduced in newborn M1 macrophages (1.4- to 1.9-fold less at baseline and 15 min after LPS stimulation; Figure 5H). No difference in IRF8 expression was noticed between newborns and adults (Figure 5I). Phospho-Akt levels were not affected by LPS stimulation in newborn and adult M1 macrophages (Figure 5J). Combined altogether, and considering that NF-κB and MAPK signaling pathways were not impaired in newborns, our data pointed toward IRF5 as a possible regulator, which decreased expression in newborn M1 macrophages could be involved in a selectively reduced TNF production.

### IRF5 Overexpression Restores TNF Secretion in Newborn M1 Macrophages

To investigate the relationship between lower levels of IRF5 and reduced TNF secretion in newborn M1 macrophages, we transduced newborn M1 macrophages with an IRF5 expressing adenoviral vector. Transduction increased IRF5 expression 1.6-fold (Figure 6A) and markedly increased (2.1- to 4.0-fold) TNF secretion, while it did not affect IL-6, IL-8, and IL-10 secretion (Figure 6B). Next, we examined the recruitment of IRF5 and RNA Pol II to the TNF promoter by ChIP. IRF5 binding was detected in unstimulated M1 macrophages and strongly decreased 1 h after exposure to LPS in both newborns and adults (Figure 6C). LPS stimulation for 1 h led to the recruitment of RNA Pol II to the TNF promoter in newborn and adult M1 macrophages. In summary, a selective increase in LPS-induced TNF production following IRF5 overexpression in newborn M1 macrophages strongly suggests an important role for IRF5 in shaping the TNF response in newborns. Yet, the mechanism of action of IRF5 might be independent of its recruitment to the *TNF* promoter following LPS stimulation.

## Discussion

**Figure 7:**
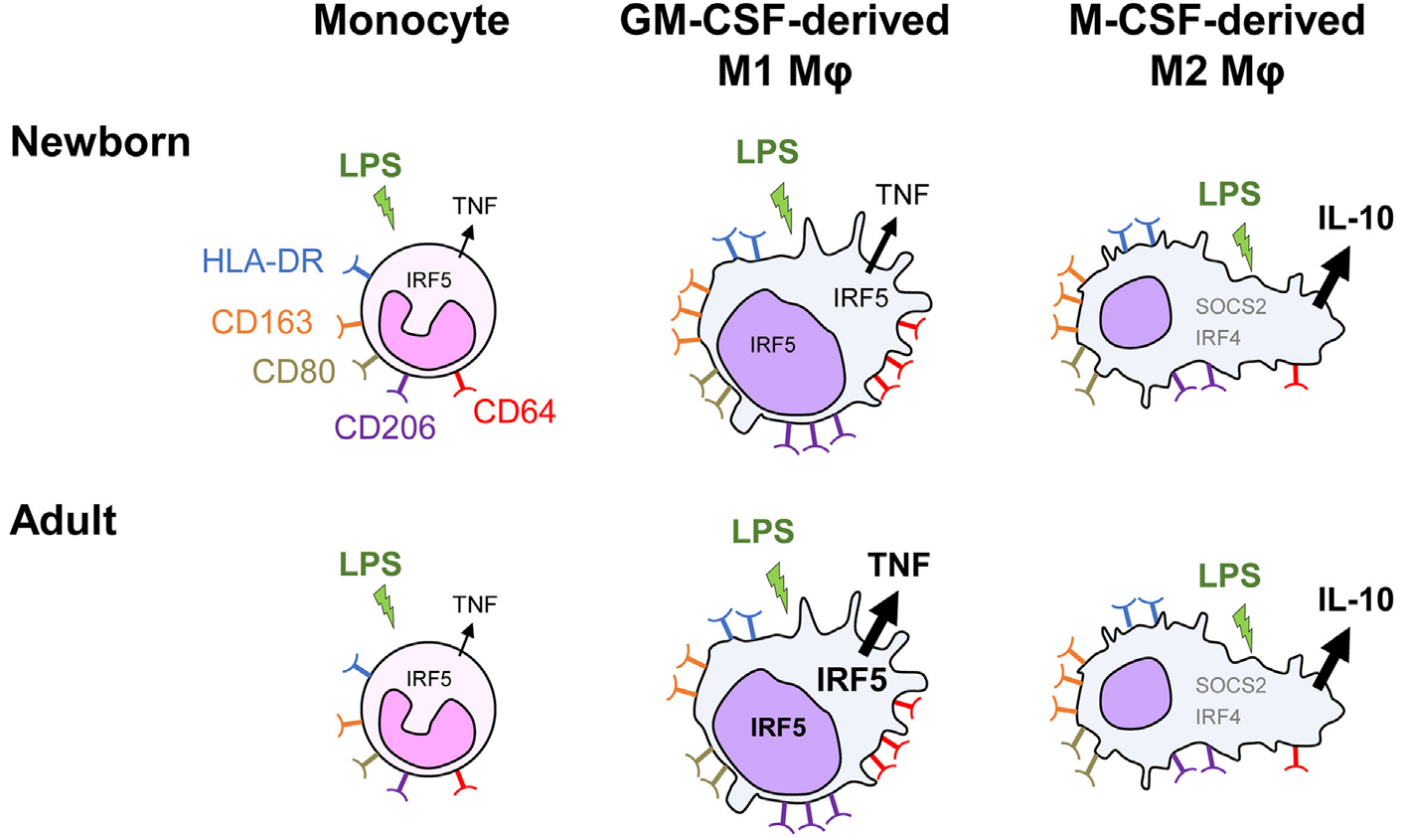
Distinct TLR4 response in neonatal monocyte-derived macrophages. Exposure of newborn and adult monocytes to GM-CSF and M-CSF drives their differentiation into macrophages having an M1 (CD64^high^ and CD206^high^ round cells) and an M2 (SOCS2^high^, IRF4^high^ and CD163^high^ elongated cells) phenotype. Macrophages express high levels of membrane-bound HLA-DR, CD80, CD163, and CD206, with newborn M1 macrophages expressing higher levels of the hemoglobin scavenger receptor CD163 than adult M1 macrophages. Following TLR4 stimulation, newborn monocytes secrete TNF to a similar level as adult monocytes. M1 macrophages secrete higher levels of TNF but low levels of IL-10, whereas M2 macrophages secrete low levels of TNF but high levels of IL-10. TNF secretion is threefold to sixfold lower in newborn than in adult M1 macrophages, while the production of other cytokines (IL-1β, IL-6, IL-8, and IL-23) is at similar levels. IRF5, a transcription factor implicated in M1 polarization, increases in the cytoplasm and in the nucleus during monocyte to M1 macrophage differentiation. Both cytoplasmic and nuclear levels of IRF5 are lower in newborn than adult M1 macrophages. IRF5 overexpression in newborn M1 macrophages restores LPS-induced TNF production in newborn M1 macrophages.

We report that monocyte-derived M1 macrophages from newborns exhibit a strongly reduced capability to release TNF upon TLR4 stimulation, while the production of other cytokines is at similar levels as in adults. IRF5 is a key factor shaping this important functional characteristic of newborn macrophages (Figure 7).

Studies in mice, rats, and monkeys have described organ, tissue, and species-specific phenotypic and functional differences between newborn and adult macrophages. Globally, newborn macrophages display reduced capacities to kill bacteria (56–59) and to produce proinflammatory cytokines (59–62) while they release anti-inflammatory cytokines at the same levels as adult macrophages (61–63). Previous studies in humans have investigated mixed populations of umbilical cord blood mononuclear cells or monocyte-derived cells, without a phenotypic characterization of differentiated cells (64–67). Cord blood-derived macrophage-like cells have a reduced capacity to kill group B *Streptococcus* and *Candida*, and release lower amounts of TNF, IL-1β, IL-6, and IL-12 in response to LPS (64, 68).

M-CSF is constitutively expressed by several cell types including fibroblasts, endothelial cells, stromal cells, and osteoblasts (69). Besides promoting survival, proliferation, and differentiation of bone marrow progenitors and monocytes, steady-state expression of M-CSF contributes to polarize macrophages toward an M2 phenotype (70). GM-CSF is expressed at low levels in the circulation and in tissues at homeostasis and plays a critical role in the terminal differentiation and functions of alveolar macrophages (71). Inflammation and infections trigger the production of GM-CSF by endothelial cells, fibroblasts macrophages, T cells, mast cells, and natural killer cells. GM-CSF drives M1 polarization, which is essential to mount efficient antimicrobial responses. Morphological and phenotypical analyses confirmed that newborn monocytes differentiate into cells adopting features of M1 and M2 macrophages, similar to adult cells. Uniquely, CD163 was strongly upregulated by both M1 and M2 macrophages in newborns, while this molecule is commonly used as an M2 marker in adults [(41, 49, 50) and our data]. Reduced activation of IRF5 in newborns might be implicated as IRF5 downregulates CD163 expression in adult macrophages (23). CD163 is a scavenger receptor involved in the clearance of free hemoglobin (72). During the neonatal period, high expression of CD163 in both M1 and M2 macrophages could be relevant, since newborn infants have an elevated turnover of erythrocytes under physiologic conditions and are prone to hemolysis during infection (8, 73, 74).

The lower capacity of newborn M1 macrophages to release TNF is most likely acquired during the process of monocyte to macrophage differentiation. Indeed, newborn monocytes released similar levels of TNF as adult monocytes under the experimental conditions used in the present study. Clearly, newborn M1 macrophages are not globally defective in TLR4 signaling, considering that TLR4 expression, NF-κBp65 nuclear translocation, ERK1/2 phosphorylation and MKP-1 expression, and production of IL-1β, IL-6, IL-8, and IL-23 are not diminished in newborn M1 macrophages.

IRF5 regulatory axis shapes the phenotype of newborn macrophages and plays an important role in systemic inflammation (54), as IRF5-deficient mice are protected from LPS-induced systemic inflammation and autoimmune diseases (75). Freshly isolated newborn monocytes expressed IRF5 to a similar extent as adult monocytes but had reduced expression and nuclear translocation of IRF5 when cultured with GM-CSF. Moreover, adenoviral-mediated IRF5 overexpression in newborn M1 macrophages restored TLR4-mediated TNF secretion, while it did not impact IL-6, IL-8, and IL-10 production, indicating that IRF5 might play a key role in the selective reduction of TNF secretion observed in newborn macrophages. In contrast, germline deletion of IRF5 impairs LPS-induced production of Th1/Th17 cytokines in mice (75), and IRF5 overexpression in adult human macrophages increases expression of TNF, IL-1β, IL-12p70, and IL-23 and reduces secretion of IL-10 (23). These data suggest that IRF5 has a broader impact on cytokine production in adult than in newborn cells. Further studies will be required to define whether IRF5 differential expression impacts on immune functions besides TNF production in newborns.

Following exposure of macrophages to LPS, IRF5 is recruited to regulatory elements of the *TNF* gene and stimulates transcription (75, 76). In adult M1 macrophages, NOD2 stimulation triggers an IRF5-dependent activation of MAPKs, NFκB, and Akt2, increasing TNF, IL-1β, and IL-12 production (77). However, in our study, LPS stimulation did not increase IRF5 expression, nuclear translocation, and recruitment to the TNF promoter in M1 macrophages. Moreover, NF-κB and MAPKs signaling pathways were not impaired in newborn M1 macrophages, and Akt was not activated following LPS stimulation. Chromatin remodeling is implicated in monocyte to macrophage differentiation and macrophage polarization (78, 79), and histone acetylation and methylation are regulators of *TNF* gene expression (80). Further studies will be required to address whether GM-CSF induced a specific epigenetic reprogramming in newborn monocytes making newborn M1 macrophages less prone to transcribe *TNF* in response to TLR4 stimulation. It will be also important to define whether posttranscriptional modifications of IRF5 required for optimal *TNF* transcription are reduced in newborn macrophages.

Previous studies have identified reduced activation of IRF family members as mechanisms underlying the limited capacity of neonatal DCs to mount proinflammatory responses. Lower IRF3 activity in newborn monocyte-derived DCs in response to TLR4 stimulation is associated with reduced expression of IFN-β, IL-12p70, and the IFN-inducible chemokines CXCL9, CXCL10, and CXCL11 (5). Moreover, the limited production of typeI/III IFNs by newborn plasmocytoid DCs exposed to herpes simplex virus-1 is linked to a reduced nuclear translocation of IRF7 (81). Combined altogether, these studies put forward a major role of IRFs in shaping the unique characteristics of newborn myeloid cells.

Our findings recognize characteristics of newborn macrophages that could be relevant to the vulnerability to infections observed during the neonatal period. Indeed, TNF is an early response cytokine that plays a crucial role in recruiting innate immune cells to sites of infection and promoting microbicidal activities. However, during established infections, excessive levels of TNF participate to the dysregulated immune responses that contribute to the pathogenesis of sepsis (74). Moreover, inflammation can cause considerable damage to developing organs, resulting in death or long-term disability (10). Thus, lower production of TNF by newborn macrophages exposed to microbial products could be advantageous to limit inflammatory responses during postnatal colonization of the skin and gastrointestinal tract and to reduce organ dysfunction and damage during systemic infection. The observation of a selective reduction in TNF secretion by newborn macrophages, while activation of major signaling pathways and production of other cytokines is maintained, supports the concept that immune responses are highly regulated to meet the specific requirements of early life. While we focused on differences between the developing neonatal immune system and the fully developed adult immune system, the absence of data from children is a limitation.

In summary, we identified distinct characteristics of the monocytic lineage in newborns that show limited IRF5 activation during monocyte to macrophage differentiation, and a specific reduction of TNF production upon TLR4 stimulation in M1 macrophages. These observations are relevant in the context of neonatal inflammation and infection and may provide a new potential target for immune modulating therapies during the neonatal period.

## Ethics Statement

This study was carried out in accordance with the recommendations of Swiss Ethics Comittees on research involving human subjects. The protocol was approved by the Cantonal Human research Ethics Committee of Vaud (CER-VD, Lausanne, Switzerland). All subjects gave written informed consent in accordance with the Declaration of Helsinki.

## Author Contributions

EG and AS designed the study and wrote the first draft of the manuscript. AS and MW performed experiments. JH performed CyTOF studies. AS, JH, MP, TC, TR, and EG analyzed and interpreted the data. All the authors revised the manuscript.

## Conflict of Interest Statement

The authors declare that the research was conducted in the absence of any commercial or financial relationships that could be construed as a potential conflict of interest.
