# Supplementary material for: IRF5 Is a Key Regulator of Macrophage Response to Lipopolysaccharide in Newborns
Source: Front Immunol. 2018 Jul 11;9:1597. doi: 10.3389/fimmu.2018.01597 (PMC6050365; doi:10.3389/fimmu.2018.01597)

Supplementary Material

IRF5 is a key regulator of macrophage response to lipopolysaccharide in newborns

Anina Schneider, Manuela Weier, Jacobus Herderschee, Matthieu Perreau, Thierry Calandra, Thierry Roger and Eric Giannoni[*]

*** Correspondence:** Eric.Giannoni@chuv.ch

Supplementary Table 1

Antibodies used for flow cytometry, Western blotting and CyTOF analyses.

Purpose	Target	Clone	Coupling or reference	Company
Flow cytometry	CD115 (M-CSFR)	9-4D2-1E4	PerCy5.5	Biolegend
	CD116 (CD116)	REA211	PE	Miltenyi Biotec
	CD14	M5E5	Pacific Blue	Biolegend
	CD16	B73.1	APC	Biolegend
	CD163	GHI/61	PE-CF594	BD Biosciences
	CD206	19.2	APC	eBioscience
	CD284	HTA125	PE	Miltenyi Biotec
	CD80	L307.4	FITC	BD Biosciences
	HLA-DR	L243	APC-H7	BD Biosciences
	SLAN	DD-1	FITC	Miltenyi Biotec
	Isotype control	REA	PE	Biolegend
	Isotype control	G155-17	APC-H7	BD Biosciences
	Isotype control	RTK2071	PerCy5.5	Biolegend
Western blot	NF-κBp65	D14E12	8242	Cell Signaling Technology
	IκBα	L35A5	4814	Cell Signaling Technology
	pERK1/2	D13.14.4E	4370	Cell Signaling Technology
	ERK1/2	137F5	4695	Cell Signaling Technology
	pp38	D3F9	4511	Cell Signaling Technology
	p38	D13E1	8690	Cell Signaling Technology
	pJNK	81E11	4668	Cell Signaling Technology
	JNK	56G8	9258	Cell Signaling Technology
	pAKT	D9E	4060	Cell Signaling Technology
	AKT	C67E	4691	Cell Signaling Technology
	STAT5	polyclonal	9363	Cell Signaling Technology
	IRF5	E1N9G	13496	Cell Signaling Technology
	IRF8	D20D8	5628	Cell Signaling Technology
	TBP	polyclonal	8515	Cell Signaling Technology
	GAPDH	polyclonal	S0011	Epitomics
	βactin	polyclonal	4967	Cell Signaling Technology
CyTOF	CD14	M5E2	160Gd	Biolegend/MaxPar
	CD45	HI30	Y89	Fluidigm
	CD45	HI30	113In	Biolegend/MaxPar/Trace Sciences*
	CD45	HI30	115In	Biolegend/MaxPar/Trace Sciences*
	NF-κBp65	K10-895.12.50	154Sm	BD Biosciences
	pERK 1/2	D13.14.4E	167Er	Fluidigm
	pp38	D3F9	156Gb	Fluidigm
	pStat1	58D6	153Eu	Fluidigm
	pStat3	4/P-STAT3	158Gd	Fluidigm
	pStat5	47	150Nd	Fluidigm
ChIP	RNA Polymerase II	D8L4Y	14958S	Cell Signaling Technology
	IRF5	E1N9G	13496S	Cell Signaling Technology

PE: phycoerythrin; FITC: fluorescein isothiocyanate; APC: allophycocyanin, PerCP: peridinin chlorophyll protein complex. *Antibody conjugated using polymer from the MaxPar kit and purified metal isotopes from Trace Sciences.

Supplementary Figure 1.

Gating strategy used to identify classical, intermediate and non-classical monocytes. Cells were selected **(A)**, aggregates were excluded **(B+C)**, and gating was performed on live cells **(D)**. CD14 was gated against CD16 to define the $CD14^{++}CD16^{-}$ classical monocytes (small square) **(E)**. SLAN was used to define the $SLAN^{-}CD14^{++}CD16^{+}$ intermediate and $SLAN^{+}CD14^{+}CD16^{++}$ non-classical monocytes **(F)**.

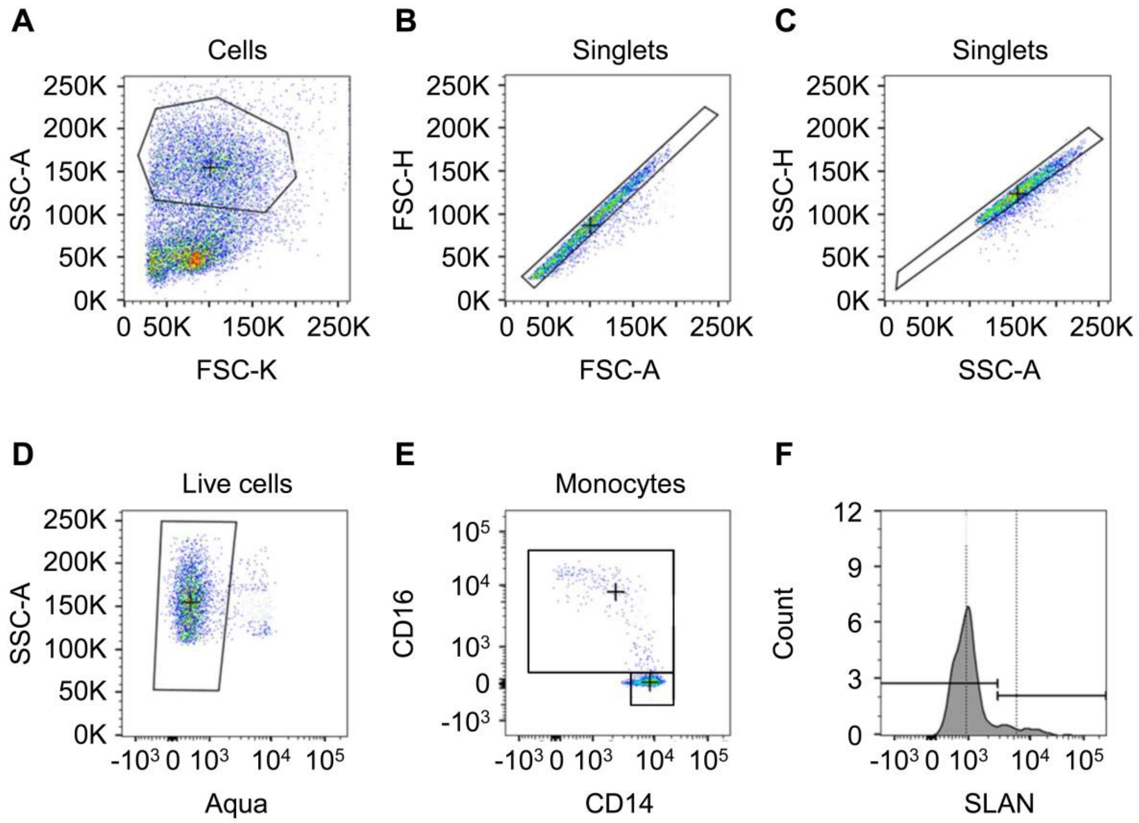

Supplement: Supplementary file 1 [file data_sheet_1.PDF]
